# The emergence of plasmid mediated quinolone resistance *qnrA*2 in extended spectrum β-lactamase producing *Klebsiella pneumoniae* in the Middle East

**DOI:** 10.1186/s40199-015-0116-7

**Published:** 2015-06-28

**Authors:** Leila Vali, Ali A. Dashti, Mehrez M. Jadaon, Sherief El-Shazly

**Affiliations:** Department of Medical Laboratory Sciences, Faculty of Allied Health, Sciences, Kuwait University, P.O. Box 31470-Sulaibekhat, 90805 Sulaibekhat, Kuwait

**Keywords:** *Klebsiella pneumoniae*, Fluoroquinolones, *qnr*, Pulsed-field gel electrophoresis, Extended spectrum β-lactamase

## Abstract

**Background:**

*Klebsiella pneumoniae* is one of the most important opportunistic pathogens causing serious complications in patients in hospitals and community. The clinical significance of *K. pneumoniae* is mainly due to its ability to acquire multiple antibiotic resistance genes. In this study we report the findings of a survey of plasmid mediated quinolone resistance in Extended-Spectrum β-lactamase (ESBL)-producing *K. pneumoniae* in Kuwait.

**Methods:**

Clinical samples were collected from the microbiology laboratories of three major hospitals. Isolates were confirmed as ESBL-producers by disc diffusion method and PCR for the presence of *bla* genes. Antimicrobial susceptibility testing and genetic analysis were performed to detect the presence of a number of genes conferring resistance to β-lactam and fluoroquinolone antimicrobial agents including *bla*_SHV_, *bla*_TEM_, *aac* (6')-*Ib-cr*, *qnrA*, *qnrB* and *qnrS*. Pulsed-field gel electrophoresis (PFGE) was used for typing the isolates.

**Results:**

In total 173 ESBL-producing *K. pneumoniae* were detected. *qnr* genes were identified in 27 (15.6 %) isolates and *aac(6′)-Ib Ib-cr* gene in 26 (96 %). One (3.7 %) contained *qnrA*2, 21 harbored *qnrB*1 (78 %) and 5 (18.5 %) contained *qnrS*. Twenty one (78 %) isolates contained all three *bla* genes. PFGE showed diverse profiles.

**Conclusion:**

We identified for the first time the emergence of the mobile fluoroquinolone resistance *qnrA*2 in a clinical isolate in the middle east and also showed the dissemination of *aac* (6')-*Ib-cr*, *qnr*B, and *qnr*S genes among ESBL-producing *K. pneumoniae* in Kuwait. The abundance of plasmid mediated resistance to fluoroquinolones among ESBL-producing *K. pneumoniae* is alarming as it facilitates therapy failure. Preventing the spread of these isolates is crucial if we are to sustain the effectiveness of the limited choices we have left in antimicrobial therapy.

## Background

Members of the *Enterobacteriaceae* family in particular the multi-drug resistant *Klebsiella pneumoniae* are amongst the opportunistic pathogens. *K. pneumoniae* causes a wide range of infections from urinary and respiratory tract infections to bacteraemia, particularly in elderly or debilitated patients. Antibiotic resistance is an important factor that hinders therapy and delays improvement in patients’ health. In *K. pneumoniae* resistance to antimicrobial agents is caused by different mechanisms however the production of extended spectrum β-lactamases (ESBLs) such as CTX-M and other enzymes including TEM-1, TEM-24, SHV-12 and the plasmid-mediated AmpC (CMY-2) are considered highly important [1–3]. CTX-M-15 producing strains can often contain variants of an aminoglycoside-modifying enzyme expressed by *aac(6′)-Ib-cr* genes. Aminoglycoside-modifying enzymes cause reduced susceptibility to aminoglycosides and to some fluoroquinolones [4]. Fluoroquinolone resistance in *K. pneumoniae* can arise by mutations in the chromosomal type II topoisomerases and changes in the expression of efflux pumps and porins. Furthermore fluoroquinolone resistance can also be facilitated by plasmid-mediated resistance determinants *qnr* that express the pentapeptide repeat proteins. These proteins protect the quinolone targets (DNA gyrase or topoisomaerase IV enzymes) from the inhibitory activity of fluoroquinolone antibiotics [5, 6]. In view of the genetic heterogeneity variants of *qnrA*, *B* and *S*-like genes share 94 % to 99 %, 85 % to 99 % and 90 % nucleotide identity respectively [7].

In addition *K. pneumoniae* has been reported to produce clinically important carbapenemases; such as Ambler class B metallo-β-lactamases (NDM), the class A enzymes (KPC) and the class D oxacillinase enzymes (OXA-48) [8, 9].

Following the rise in the use of cephalosporins in hospitals; the incidence of infections caused by ESBL-producing *K. pneumoniae* has soared [2, 10]. Resistance to 3^rd^ generation cephalosporins is predominantly associated with the presence of *bla*_CTX-M_ genes located on plasmids [11]. Therefore plasmid mediated resistance to fluoroquinolones among ESBL producing *K. pneumoniae* is alarming as it facilitates therapy failure. Moreover; the exchange of the genetic information among bacterial species threatens the efficacy of fluoroquinolones and increases the dependency on carbapenem antibiotics for treatment. The purpose of the present study was to determine the prevalence of plasmid-mediated fluoroquinolone resistance in ESBL-producing *K. pneumoniae* in hospitalized patients in Kuwait.

## Material and methods

### Bacterial isolates

In-patient clinical samples were collected from the microbiology laboratories of three major governmental hospitals that serve the six governorates of Kuwait, namely Al-Ahmadi hospital, Al-Amiri hospital and Adan hospital. All three hospitals are tertiary health care providers with bed capacities of 300, 500 and 600, respectively. The average number of specimens processed every day in each hospital varies from 500 to 700 which includes samples from out-patient and in-patient specialists units. A table was created based on the patients’ records containing information such as age, gender, hospital, and type of specimen. Specimens were processed by clinical staff members of the diagnostic microbiology laboratories using standard protocols. The species identification was carried out by the API (bioMérieux, Marcy l’Etoile, France) and the VITEK 2 systems (Vitek AMS; bioMérieux Vitek Systems Inc., Hazelwood, MO, USA). The results were analyzed according to the Clinical and Laboratory Standards Institute (CLSI) (2012) guidelines [12]. The isolates were stored in 10 % skim milk at −70 °C.

### Susceptibility testing

Antibiotic susceptibility tests were performed by the Kirby-Bauer method disc diffusion test following the CLSI (2012) criteria and recommendations [12]. The antibiotics tested were ampicillin, ampicillin/sulbactam, amoxicillin, amoxicillin/clavulanic acid, piperacillin/tazobactam, ceftazidime, cefepime, ceftriaxone, cefazolin and cefuroxime. The Minimum Inhibitory Concentration (MIC) was determined by agar dilution method and E-test when available for the following antibiotics: trimethoprim gentamicin, cefotaxime, imipenem, meropenem, ciprofloxacin, levofloxacin and tetracycline. Isolates that showed resistance to at least three classes of antibiotics were considered as MDR. Isolates that were detected as resistant to cefoxitin were further investigated for the presence of an *amp*C β-lactamase by using multiplex PCR [13, 14].

### Double-disc synergy method

ESBLs were detected as previously described using the disc approximation and double-disc synergy methods and confirmed with cefotaxime and ceftazidime E-test ESBL strips (AB Biodisk, Biomerieux-diagnostics, Durham, NC, USA) [2]. For the disc approximation test, clavulanate diffusion from an amoxicillin–clavulanate (AMC30) disc was used to test for synergy with cefotaxime, ceftazidime, cefuroxime, cefepime and cefixime (Oxoid) as described previously [15]. For the double-disc synergy test, a ceftazidime disc (30 μg) was placed 30 mm away from a disc containing amoxicillin–clavulanate (60/10 μg). ESBL production was considered positive when an enhanced zone of inhibition was visible between the β-lactam and β-lactamase inhibitor-containing discs. For the E-test, ESBL strips containing ceftazidime and ceftazidime–clavulanate and strips containing cefotaxime and cefotaxime–clavulanate were used to determine the MIC ratio according to the manufacturer's instructions (AB Biodisk, Biomerieux-diagnostics, Durham, NC, USA). Cultures were incubated aerobically at 37 °C for 18–24 h. CTX-M-15 β-lactamase enzyme displays a catalytic activity toward ceftazidime.

### Detection of *bla* genes and other resistance determinants

The presence of resistant genes (listed below) were investigated by PCR assays as previously reported [16]. PCR was conducted in a GeneAmp 9700 (Perkin-Elmer, Illinois, USA) system using the conditions specified for each primer; corresponding to the source references. The resistant genes investigated were *bla*_TEM-1_, *bla*_SHV_, *bla*_CTX-M-like_, *bla*_NDM_, *bla*_OXA-1_, *qnr*A, *qnr*B, *qnr*S, *aac(6′)-Ib Ib-cr*, *gyr*A, *par*C, *gyr*B, *par*E, *intI*1, *intI*2, *bla*_KPC_, *bla*_VIM_, *bla*_IMP_, *bla*_OXA-48,_ and *amp*C. The detection of *bla*_PER_, *bla*_GES_ and *bla*_VEB_ was performed by PCR according to Opazo *et al.* [17].

Isolates resistant to ciprofloxacin and for which the ceftaxidime or cefotaxime MICs were >8 mg/L were screened for ESBLs and *qnr* genes. Double disc and combination disc tests were used for ESBLs confirmation.

Amplified PCR products were purified with Qiagen purification kit (Qiagen, Limburg Netherlands) according to the manufacturer's instructions and both strands were sequenced by automated ABI3100 DNA sequencer (Applied Biosystems, Foster City, CA, USA). The BLAST program of the National Centre for Biotechnology Information (http://www.ncbi.nlm.nih.gov) was used to search and compare databases for similar nucleotide sequences.

### Pulsed-Field Gel Electrophoresis (PFGE)

PFGE was performed by using the restriction enzyme *Xba*I [18], with a run time of 20 h and switch times of 5 to 50 and 5 to 35 s at 14 °C and 6 V/cm (CHEF-DR II System; Bio-Rad, Hercules, CA, USA). The PFGE profiles were analyzed by using BioNumerics software version 7.1 (Applied Maths, Sint-Martens-Latem, Belgium). Statistics was added at the end of the methods section.

### Statistical analysis

Out of the total number of isolates included hereby, the percentage of the different ESBL-producing bacteria were calculated in each of the three hospitals participating in the study.

DNA profiles were analysed by the unweighted pair method with arithmetic average (UPGMA) using BioNumerics v.7.1. The development of the algorithms necessary for the comparison of fingerprinting profiles of isolates was based on the Dice correlation coefficient. The hierarchic Cluster analysis and phylogenetic trees were subsequently analysed with an optimization of 1.0 % and a tolerance of 1 %. Isolates were considered to belong to the same PFGE clone if their Dice similarity index was ≥85 %.

## Results

### Bacterial isolates

All three governmental hospitals participated during our study period from 2010 to 2012; however there were inconsistencies in the level of strain contribution from each hospital. Therefore under-representation of ESBL-mediated *K. pneumoniae* might exist. A total of 832 ESBL-producing *Enterobacteriaceae* isolates were obtained Table 1. They comprised of 606 *Escherichia coli* (73 %), 11 *Enterobacter cloacae* (1.3 %), 28 *Proteus mirabilis* (3 %), 14 *Klebsiella oxytoca* (1.7 %) *and* 173 *Klebsiella pneumoniae* (21 %).

**Table 1:** The total number of ESBL producing *Enterobacteriaceae* isolates containing *qnr* genes during this study

Hospital	Enterobacteriaceae (ESBL producers)	*Escherichia coli*	*Enterobacter cloacae*	*Proteus mirabilis*	*Klebseilla oxytoca*	*Klebsiella pneumoniae*	*qnr* positive *K. pneumoniae*
Amiri	480	383 (80 %)	1 (0.2 %)	18 (3.8 %)	6 (1 %)	72 (15 %)	5 (6.9 %)
KOC	137	83 (61 %)	6 (4 %)	7 (5 %)	4 (3 %)	37 (27 %)	10 (2.7 %)
Adan	215	140 (65 %)	4 (1.8 %)	3 (1.4 %)	4 (1.8 %)	64 (30 %)	12 (18.8 %)
Total	832	606 (73 %)	11 (1.3 %)	28 (3 %)	14 (1.7 %)	173 (21 %)	27 (15.6 %)

The antibiotic sensitivity testing for *K. pneumoniae* isolates was performed followed by double-disc synergy tests. The results were confirmed with PCR and sequencing the product.

From the total of 173 (21 %) ESBL-producing *K. pneumoniae qnr* genes were identified in 27 (15.6 %) isolates. The distribution of the isolates is presented in Table 2.

**Table 2:** The list of K. pneumoniae isolates

Hospital	Isolate number	Specimen	Gender	Age	Species
Adan	ADA-17	Urine	M	NA	K. pneumoniae
	ADA-28	Urine	M	NA	K. pneumoniae
	ADA-30	Urine	M	NA	K. pneumoniae
	ADA-39	Bile	F	33	K. pneumoniae
	ADA-45	Urine	M	76	K. pneumoniae
	ADA-49	Bile	F	33	K. pneumoniae
	ADA-69	Urine	F	78	K. pneumoniae
	ADA-89	Urine	M	84	K. pneumoniae
	ADA-111	Urine	M	77	K. pneumoniae
	ADA-140	Urine	F	77	K. pneumoniae
	ADA-214	Urine	M	79	K. pneumoniae
	ADA-215	Urine	F	8	K. pneumoniae
Ahmadi	KOC-2	Urine	M	82	K. pneumoniae
	KOC-7	Urine	F	47	K. pneumoniae
	KOC-12	Catheter Tip	F	14	K. pneumoniae
	KOC-32	Urine	F	36	K. pneumoniae
	KOC-37	Urine	F	36	K. pneumoniae
	KOC-63	Urine	F	90	K. pneumoniae
	KOC-64	Urine	F	58	K. pneumoniae
	KOC-66	Catheter Tip	F	72	K. pneumoniae
	KOC-74	Urine	F	60	K. pneumoniae
	KOC-105	Diabetic Foot	M	59	K. pneumoniae
Al-Amiri	Y-2	Diabetic Foot	M	52	K. pneumoniae
	Y-4	Urine	M	78	K. pneumoniae
	Y-12	Bedsore Swab	F	65	K. pneumoniae
	Y-15	Penile Swab	M	81	K. pneumoniae

*NA* Not available

### Sensitivity to antimicrobial agents

All the 27 *qnr* positive isolates were resistant to ampicillin (MIC > 16), ciproflaxoacin (MIC > 4), cefotaxime (MIC > 4), ceftazidime (MIC > 4), ceftriaxone (MIC > 4), and gentamicin (MIC > 16); but sensitive to imipenem (MIC > 1) and meropenem (MIC > 1).

Sequencing the PCR products showed the distribution of the resistance determinants (Table 3). *bla*_CTX-M-15_ was detected in 25 (93 %), *bla*_CTXM-2_ in 2 (7 %), *bla*_TEM_ in 21 (78 %), *bla*_SHV_ and *aac(6′)-Ib Ib-cr* gene was found in 26 (96 %) isolates. Twenty one (78 %) isolates contained all three *bla* genes. From the 27 *K. pneumonia* isolates only 1 (3.7 %) contained *qnrA2*, 21 harbored *qnrB*1 (78 %) and 5 (18.5 %) contained *qnrS*. Class I integrons (*intI*1) was detected in 25 (93 %) isolates but class II integrons (*intI*2) was not found in any of the isolates tested. *bla*_KPC_, *bla*_VIM_, *bla*_PER_, *bla*_GES_, *bla*_VEB_, *bla*_IMP_, *bla*_OXA1_, *bla*_OXA48_ and *amp*C were not detected in this study. No mutations were found in *gyr*A and *par*C.

**Table 3:** The profiles of *qnr* positive *K. pneumoniae* isolates and other resistance genes studied

Profiles of the antibiotic resistance genes	No. of isolates
*qnr*B1, *bla*_CTX-M-15_	2
*qnr*B1, *bla*_CTX-M-2_, *bla*_SHV_ *, aac(6*′*)-Ib-cr*	1
*qnr*B1, *bla*_CTX-M-15,_ *bla*_SHV_ *, aac(6*′*)-Ib-cr*	3
*qnr*S, *bla*_CTX-M-2_, *bla*_TEM-1,_ *bla*_SHV_ *, aac(6*′*)-Ib-cr*	1
*qnr*S, *bla*_CTX-M-15_, *bla*_TEM-1,_ *bla*_SHV_ *, aac(6*′*)-Ib-cr*	4
*qnr*A2, *bla*_CTX-M-15_, *bla*_TEM-1_, *bla*_SHV_ *, aac(6*′*)-Ib-cr*	1
*qnr*B1, *bla*_CTX-M-15_, *bla*_TEM-1,_ *bla*_SHV_ *, aac(6*′*)-Ib-cr*	15
Total	27

### PFGE

**Fig. 1:**
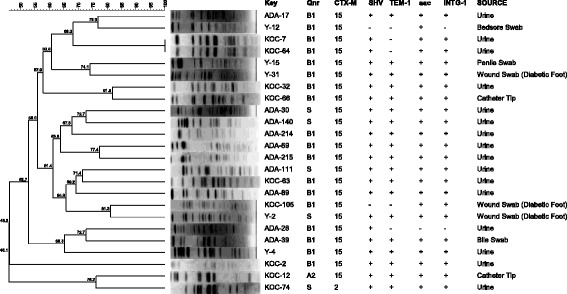
PFGE showing the relationship between DNA banding patterns

PFGE showed diverse profiles among the isolates tested (Fig. 1). Only two isolates (KOC 7 and KOC 64) from urinary tract infection had identical PFGE patterns. The majority of isolates with similar *qnr* genes; displayed similar antimicrobial resistance phenotypes but their PFGE profiles were not identical. In total three isolates (ADA-45, ADA-49 and KOC-37) were not typable by PFGE.

## Discussion

In the present study we detected a similar rate of ESBL-producing *K. pneumoniae* (21 %) as previously reported (28 %) [19]. However, we found 15.6 % (27/173) of ESBL-producing *K. pneumoniae* contained a plasmid mediated *qnr* gene. Previous research undertaken in Kuwait in 2007 did not identify either *qnr*A or *qnr*S in ESBL-producing Enterobacteriaceae and reported a low prevalence of 4.7 % (3 out of 64 isolates) of *qnrB* in *E. coli* [7]. Here we identified *qnr A*, *B* and *S* in ESBL-producing *K. pneumonia.* The incidence rate of 15.6 % *qnr* in Kuwait compared to other countries in Asia such as Iran (30.4 %) [20]. Thailand (34.6 %) [21], Malaysia (37 %) [22], India (67 %) [23] and Saudi Arabia (44 %) [24] is considerably lower.

The key findings in our study are the emergence of *qnrA*2 in the Middle East for the first time and also the spread of *qnrS* among the clinical ESBL-producing *K. pneumonia* isolates in Kuwait. *qnrA* is the first plasmid-mediated quinolone resistance identified however, in *K. pneumoniae* it is not as widespread as *qnrB* [25]. *qnrA* confers resistance to quinolones such as nalidixic acid and increases minimum inhibitory concentration (MIC) values of fluoroquinolone up to 20-folds [26]. It has also been shown that when both *qnrA* and *aac- (6`)-Ib-cr* are present in the same cell, the level of ciprofloxacin resistance is increased fourfold more than that conferred by *qnrA* alone [5]. Therefore the detection of *qnrA*2 gene in an ESBL producing *K. pneumoniae* from the catheter tip of a 14 year old female patient was alarming and a challenge for empirical antibiotic therapy such as fluoroquinolones in clinical settings. The rise of plasmid-mediated fluoroquinolone resistance is concerning for antimicrobial treatment of *K. pneumoniae* whereby the only option left will be carbapenems.

In this study *bla*_CTX-M-15_ was the predominant β-lactamase and in 93 % of the cases (25 out of 27 isolates) it was associated with the presence of *aac(6)-1b-cr*. We also identified *qnr*B1 and *qnr*S in isolates harbouring *aac(6′)-Ib Ib-cr* and *bla*_CTX-M_. These isolates were resistant to penicillins, most cephalosproins, β-lactamase inhibitors as well as fluoroquinolones posing a risk to combination β-lactam/β-lactamase inhibitor therapy. The correlation between ESBL and ciprofloxacin resistance is an indication of the important contribution of the plasmid-mediated resistance to fluoroquinolone resistance in this region.

Urine samples comprised 67 % (18/27) of the specimens followed by wound swabs 15 % (4/27). We did not find any correlation between antimicrobial resistance characteristics or specimen type and PFGE profiles. In fact overall the PFGE profiles of the tested isolates were diverse implying that the spread of *qnr* genes is not concentrated to only a specific clonal lineage. A detailed longitudinal study is required to identify the clonal identity of ESBL-producing *qnr*-positive *K. pneumonia* isolates.

## Conclusion

This manuscript reports the genotypic variation among strains of *qnr* positive K. pneumonieae in hospitalized patients in Kuwait. The presence of *qnr* and *aac-(6`)-Ib-cr* genes especially when they co-exist in ESBL-producing isolates contribute to the increase in fluoroquinolone resistance.

In conclusion the clinical importance of our findings is that the circulating MDR plasmids that contain *qnr*, *aac-(6`)-Ib-cr* and *bla* genes are concerning in hospitals as they increase the chances of *K. pneumoniae* adaptability to environmental pressures such as antibiotics. If guidelines are not in place regarding the strict use of antibiotics we will face antimicrobial therapy failure in hospitalized patients. Continuous monitoring of plasmid mediated fluoroquinolone ESBL-producing *Enterobactericeae* in clinical settings is crucial.

## Abbreviations

ESBL: Extended-Spectrum β-lactamase
*K. pneumoniae*: *Klebsiella pneumoniae*
*qnr*: Plasmid-mediated quinolone resistance gene
*bla*: β-lactamase gene
PFGE: Pulsed-field gel electrophoresis
MIC: Minimum Inhibitory Concentration
MDR: Multi-drug Resistant
PCR: Polymerase Chain Reaction
AMC: Amoxicillin/Clavulanic acid
